# Association of the gut microbiome with fecal short-chain fatty acids, lipopolysaccharides, and obesity in young Chinese college students

**DOI:** 10.3389/fnut.2023.1057759

**Published:** 2023-04-17

**Authors:** Baokuo Song, Kexin Zhao, Shuaikang Zhou, Yuling Xue, Han Lu, Xianxian Jia, Shijie Wang

**Affiliations:** ^1^College of Foods Science and Biology, Hebei University of Science and Technology, Shijiazhuang, China; ^2^Shijiazhuang People’s Hospital, Shijiazhuang, China; ^3^Junlebao Dairy Group Co., Ltd., Shijiazhuang, Hebei, China; ^4^Basic Medical College, Hebei Medical University, Shijiazhuang, China

**Keywords:** young college students, obesity, gut microbes, short-chain fatty acids, lipopolysaccharides (LPS)

## Abstract

**Introduction:**

Obesity is a growing health problem among young people worldwide and is associated with gut conditions. This study aimed to explore the relationship between obesity, intestinal microbiota, fecal short-chain fatty acids (SCFAs), and lipopolysaccharide (LPS) in young college students.

**Methods:**

16S rRNA gene sequences, SCFA and LPS contents, and obesity status were analyzed in 68 young college students (20-25 years old).

**Results:**

There were significant differences in intestinal microbial beta diversity among students with different body mass index (BMI). The abundance and proportion of Firmicutes and Bacteroides had no significant correlation with BMI. The contents of butyric acid and valeric acid in the feces of obese students were low, and the content of SCFAs had no significant correlation with BMI and LPS. The content of LPS in the feces of obese people was significantly higher than that in healthy people, and there was a significant positive correlation between LPS content and BMI.

**Conclusion:**

In general, there was a correlation between intestinal microbiota, SCFA, LPS, and BMI in young college students. Our results may enrich the understanding of the relationship between intestinal conditions and obesity and contribute to the study of obesity in young college students

## 1. Introduction

Currently, the WHO estimates that 13% of adults aged 18 and above are obese, with the number of obese people of all ages increasing worldwide (1). In China, the latest survey (2015–2019) revealed 34.3 and 16.4% of adults (≥18) to be overweight and obese, respectively (2). As attention has been mainly focused on obesity in older adults and children, it is necessary to pay attention to this condition in other important age groups (e.g., college students) (3). College students (20–25 years) are an important population vulnerable to major lifestyle changes during social and physical development (4). The incidence of obesity during this college period is reportedly high and often persist throughout adulthood (5). Obesity is the result of the complex interactions among many factors, such as the environment, social factors, and physical activity (6). According to a survey, sporting activity among college students is very low (7). In a global survey of 23 different countries, between 21.9 and 80.6% of university students were physically inactive (8). Moreover, college students are a group with long-term sedentary characteristics, which has been confirmed to increase the risk of obesity, diabetes, and other diseases (9, 10). Previous studies have shown that a high fat diet causes intestinal microbial changes, reduces fecal short-chain fatty acid (SCFA) content, and causes lipid metabolism disorders in mice (11). In addition, in relevant human studies, obesity has also been found to be related to intestinal conditions (12, 13).

It is known that the human gut microbiota contains an estimated 10^13^ cells of bacteria, which represent about 300–1,000 different taxonomic species (12). The gut microbiome has been found to be associated with obesity and metabolic disorders, and altered body mass index (BMI) could change the fecal microbial composition and abundance (14). However, the conclusions of these studies on the commonalities and differences in gut microbes in different BMI populations are still inconsistent (15, 16). On the other hand, gut microbes can affect obesity through SCFAs. Increases in intestinal permeability to bacterial lipopolysaccharide (LPS) could lead to an increase in the level of LPS in serum, which might aggravate chronic inflammation and obesity (17, 18). However, there is insufficient evidence regarding the underlying mechanism and relationship among the gut microbiome, metabolites, and obesity, warranting further investigation.

Short-chain fatty acids act as messengers between gut microbes and hosts, directly regulating host metabolic health by engaging in a range of tissue-specific mechanisms related to appetite regulation, energy expenditure, glucose homeostasis, and immune regulation (19, 20). However, the interaction between the microbiota and the number and proportion of SCFAs and their role in obesity is very complex (21). Butyrate, for example, can affect the occurrence of obesity through a variety of mechanisms, but the effect in obese individuals is still controversial (22).

Additionally, LPS is an inflammatory molecule produced in the cell membrane of gram-negative bacteria. The gut microbiota is a huge reservoir of LPS (23). LPS alters the intestinal epithelial tight junction proteins (ZO-1 and occludens) in the intestinal epithelium, and is transported from the lumen of the digestive tract into the bloodstream, aggravating chronic inflammation (24, 25). LPS is a strong activator of toll-like receptor 4 (TLR4). Binding of LPS to TLR4 activates a wide range of cell signaling pathways and induces inflammatory responses (26). LPS is absorbed by chylomicrons formed by saturated dietary fats. This step promotes inflammation in the host, thereby inducing insulin resistance. This change, in turn, affects the mechanisms by which local and systemic inflammation controls metabolic abnormalities (27, 28).

Studies on the relationship among SCFA, LPS, and obesity development are mostly based on animal models, and thus more human studies are needed. In this study, college students from Shijiazhuang (the living and eating habits of residents here are representative of those of small-to-medium-sized cites in North China) were selected as research subjects to explore the correlation among intestinal microbes, SCFAs, and LPS. Fecal samples of 68 young college students were obtained. SCFA and LPS contents in the feces and the correlations between these parameters and body indicators were evaluated to explore the metabolic differences between young college students in different weight classes. Taken together, our study provides a theoretical basis for preventing obesity among college students.

## 2. Materials and methods

### 2.1. Volunteer recruitment

College students in Shijiazhuang City, Hebei Province were recruited. A total of 68 volunteers (37.7% female), aged 22–26 years were recruited. The questionnaire included basic personal information, such as height, weight, history of chronic diseases, exercise, and recent defecation. The exclusion criteria were as follows: taking probiotics, prebiotics, or antibiotics within 3 months; (2) metabolic diseases or functional disorders; (3) heavy recent drinking, alcohol dependence, or drug dependence within 5 years; and (4) recent gastrointestinal surgery. The National Health Industry Standard of the People’s Republic of China adult weight classification was used as the standard for judging obesity. Individuals with BMI > 28.0 were assigned to the obese group (OB), those with 18.5 < BMI < 24 were assigned to the normal body weight (NW) group, and individuals with BMI < 18.5 were classified as thin weight (TW). All volunteers had no dietary restrictions. Fecal samples were collected by volunteers who were provided details of the study before sampling and were provided sterile containers for collection. The fecal samples were stored immediately after collection at −80°C.

Height and weight were measured using an ultrasonic height and weight meter. Waist circumference and hip circumference were measured with a tape measure. The equipment was calibrated before the measurement, and the inspector was able to operate the equipment skillfully. The body weight measurement required volunteers to take off their shoes and socks. The weight measurement value was accurate to 0.01 kg; the height, waist circumference, and hip circumference measurement values were accurate to 0.01 cm. BMI was calculated as weight (kg)/square of height (M2). All equipment was calibrated at the beginning of the study.

### 2.2. Fecal SCFA quantification

Short-chain fatty acids in fecal samples were quantitatively assessed as described previously (29) using a GC external standard calibration method. At least 0.2 g of fecal sample was weighed, diluted with pure water at a ratio of 1:4, and thoroughly homogenized. Then, 12 mM hydrochloric acid was used to adjust the pH value of the sample to between 2.6 and 2.8. Each sample was centrifuged at 4°C and 13,680 *g* for 15 min. The supernatant was collected and filtered using a polyethersulfone needle filter membrane. The mixture (0.5 μL) was injected into a gas chromatographer with flame ionization detection (GC-FID; Agilent, Santa Clara, CA, United States) equipped with a fused silica capillary column (DB-FFAP, 30 m × 0.25 mm × 0.25 μm; Agilent). Injection was performed using an autosampler (G4567A); 0.5 μL of sample was 10:1 injected split with a ratio of 10:1 at 230°C. Nitrogen (99.99%) was used as carrier gas at a constant flow of 2.80 mL/min. The GC temperature program was set as follows: initial temperature of 75°C, linear increase at 20°C/min to 180°C, constant for 1 min, followed by a linear increase at 50°C/min to 220°C, constant for 3 min, linear increase at 25°C/min to 250°C, constant for 2 min.

### 2.3. Fecal LPS assay

Fecal samples of at least 50 mg were prepared at 10% in PBS (pH = 7.2–7.4, concentration 0.01 mol/L). The sample temperature was maintained at 2–8°C and samples were homogenized fully. After centrifugation for about 20 min (2,000–3,000 rpm/min), the supernatant was collected. After sub-packing, one piece was used for testing and the rest was frozen and set aside. Standard, blank, and sample wells (three each) were set, and 50 μL of different concentrations of the standard were added to the standard wells. The sample diluent of 40 μL was first added to the sample hole on the enzyme-coated plate, and then 10 μL of the sample was tested (5-fold dilution). HRP-Conjugate reagent (100 μL) was added to each well, except the blank well. After closing the plate using the Closure plate membrane, samples were incubated for 60 min at 37°C. The closure plate membrane was opened, the liquid was discarded, samples were dried, washing buffer was added to every well for 30 s and then drained (repeated five times), and samples were dried by patting. Chromogen Solution A (50 μL) and Chromogen Solution B were added to each well, followed by incubation in the dark for 15 min at 37°C. Stop Solution (50 μL) was added to each well (a color change from to yellow indicated that the reaction stopped). Taking the blank well as zero, absorbance was read at 450 nm within 15 min of added the Stop Solution.

### 2.4. Genomics DNA extraction

The microbial community DNA was extracted using MagPure Stool DNA KF kitB (Magen, China) following the manufacturer’s instructions. DNA was quantified with a Qubit Fluorometer by using Qubit dsDNA BR Assay kit (Invitrogen, United States), and the quality was checked by running aliquot on 1% agarose gel.

### 2.5. Sequencing and bioinformatics analysis

Variable regions V3–V4 of bacterial 16S rRNA gene was amplified with degenerate PCR primers, 341F (5′-ACTCCTACGGGAGGCAGCAG-3′) and 806R (5′-GGACTACHVGGGTWTCTAAT-3′). Both forward and reverse primers were tagged with Illumina adapter, pad and linker sequences. PCR enrichment was performed in a 50 μL reaction containing 30 ng template, fusion PCR primer, and PCR master mix. PCR cycling conditions were as follows: 94°C for 3 min, 30 cycles of 94°Cfor 30 s, 56°C for 45 s, 72°C for 45 s, and final extension for 10 min at 72°C for 10 min. The PCR products were purified with AmpureXP beads and eluted in Elution buffer. Libraries were qualified by the Agilent 2100 bioanalyzer (Agilent, United States). The validated libraries were used for sequencing on Illumina MiSeq platform (BGI, Shenzhen, China) following the standard pipelines of Illumina, and generating 2 × 300 bp paired-end reads.

### 2.6. Statistical analysis

The alpha diversity of the gut microbiota was assessed by the Chao1 index, the number of taxa, and the Shannon diversity index, and richness and evenness analyses were conducted using the diversity function of the vegan package in R.^1^ Statistical tests were performed using IBM SPSS Statistics. Linear discriminant analysis (LDA) effect size (LEfSe) and beta diversity analyses were performed using the BGI microbial amplicon analysis platform.^2^ For variables with a normal distribution (Shapiro–Wilk test) and homoscedasticity (Levene test), one-way ANOVA was followed by *post hoc* LSD tests. In the remaining cases (variables showing a non-normal distribution), the Kruskal–Wallis test and PERMANOVA were applied. A value of *p* of <0.05 was considered significant. Microbial signatures used to distinguish specific microbiota were determined using the LEfSe method, with an alpha cutoff of 0.05 and effect size cutoff of 2.0.

## 3. Results

### 3.1. Information on volunteers

In this study, 68 young college students (42 men and 26 women) with an average age of 24 ± 2.2 years old in Shijiazhuang were recruited. Individuals with BMI > 28.0 were assigned to the obese group (OB; 36 people), those with 18.5 < BMI < 24 were assigned to the normal body weight (NW) group (22 people), and individuals with BMI < 18.5 were classified as thin weight (TW; 10 people). The subjects did not take antibiotics or other gastrointestinal stimulants. The subjects had no significant changes in body weight over 3 months and had no plans to lose or gain weight (Table 1).

**Table 1:** Physical indicators of volunteers.

	NW	OB	TW
People	22	36	10
Basic information			
Weight(kg)	59.34 ± 6.41	105.29 ± 18.07	49.53 ± 7.15
BMI	21.50 ± 1.18	33.91 ± 4.20	17.67 ± 0.96
Waistline	75.08 ± 5.69	107.29 ± 11.70	68.50 ± 6.52
Hip circumference	93.63 ± 4.47	118.58 ± 9.40	88.46 ± 4.65
Waist to hip ratio	0.80 ± 0.05	0.91 ± 0.09	0.77 ± 0.07
Body fat percentage	19.98 ± 8.53%	26.94 ± 5.36%	16.07 ± 11.19%

### 3.2. Fecal microbiome in young college students

**Figure 1:**
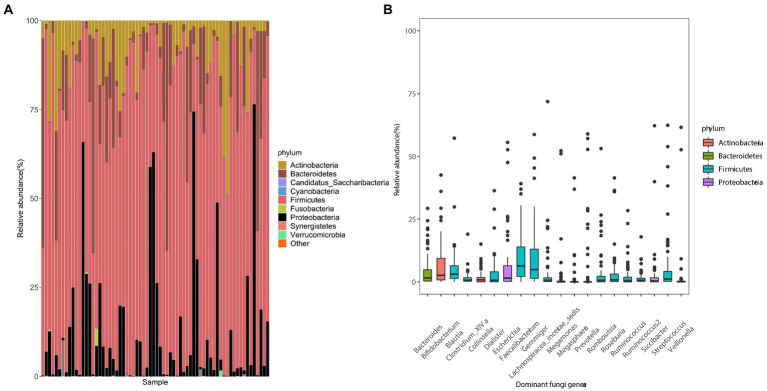
Composition of gut microbes and the average abundances of dominant bacterial genera in young adults. **(A)** Composition of the gut microbiota at the phylum level. **(B)** Composition of dominant bacterial genera (mean relative abundance >0.1%). The color corresponds to the name of each species at the particular taxonomic level and the width of the color block indicates the relative abundance.

In the fecal samples of college students, the gut microbiota was dominated by two bacterial phyla, Firmicutes and Bacteroidetes, followed by Actinobacteria, Proteobacteria, and others. This composition of the intestinal flora is common in humans (Figure 1A). The proportions of Cyanobacteria and Fusobacteria in the gut of some subjects were relatively high. Twenty dominant genera with a mean relative abundance of >1% were detected, including *Faecalibacterium* and *Gemmiger* (Figure 1B).

### 3.3. Differences in intestinal microorganisms with respect to the degree of obesity

**Figure 2:**
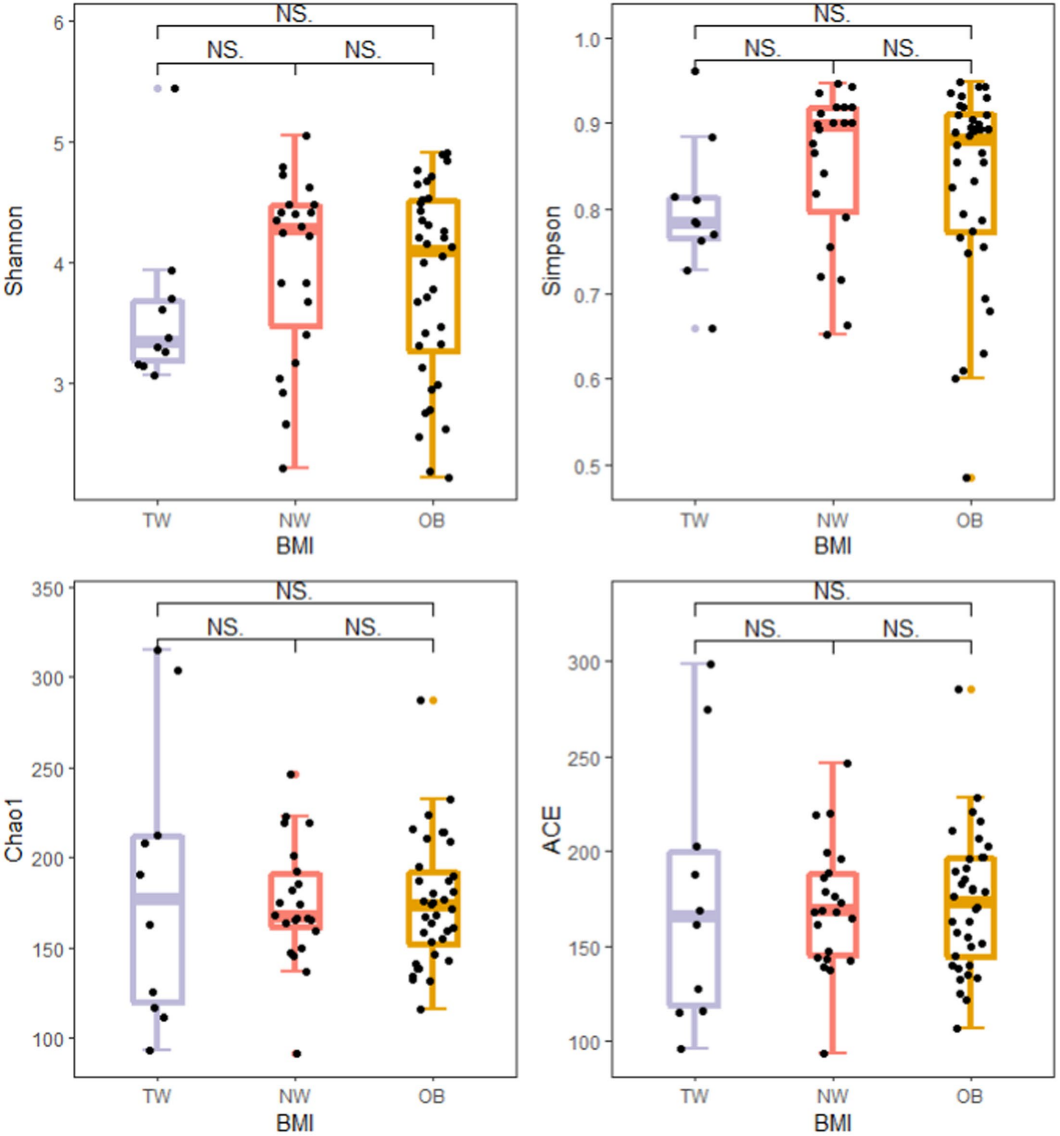
Comparison of alpha diversity indexes among groups. Alpha diversity of the gut microbiota was analyzed using the ggplot2 package and ggsignif package in R v4.1.3. *t*-test was used to test for significant differences in alpha diversity. ^NS^*p* > 0.05, ^*^p ≤ 0.05, and ^**^p ≤ 0.01.

**Figure 3:**
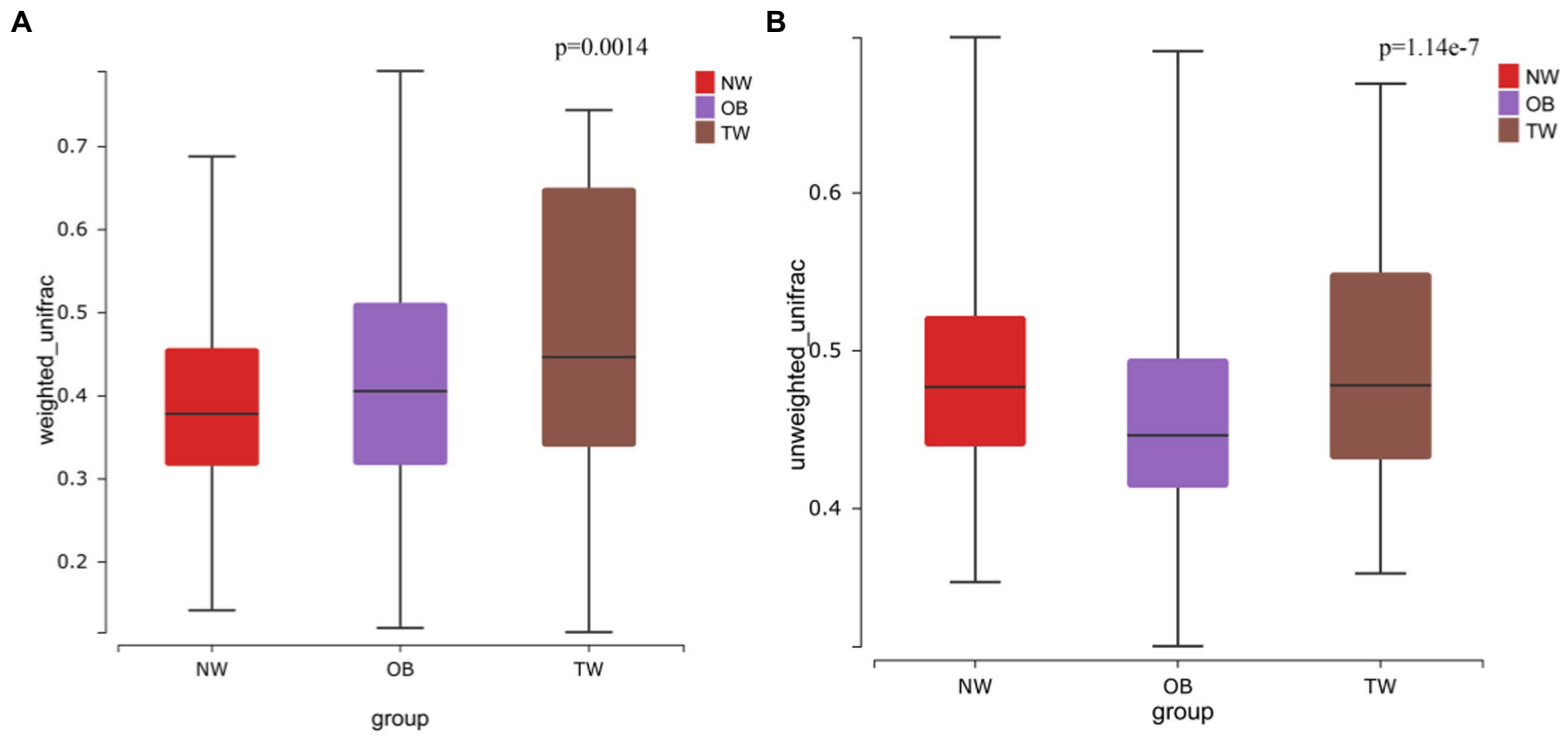
Beta diversity in three groups. **(A)** Beta diversity in the case of weighted species taxonomic abundance information; **(B)** Beta diversity in the case of weighted species taxonomic abundance information. The value of *p*s for comparisons are shown above the boxes. Kruskal–Wallis was used to test for significant differences in beta diversity.

**Figure 4:**
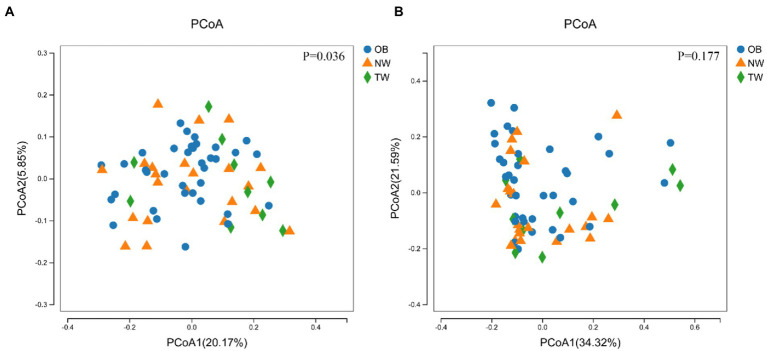
PCoA of three groups. **(A)** PCoA with weighted species taxonomic abundance information; **(B)** PCoA without weighted species taxonomic abundance information. The value of ps are shown above the boxes. In statistics, PERMANOVA was used to test for significant differences in PCoA.

To evaluate differences in the composition of the gut microbiota among groups with different BMI, we performed diversity analyses based on the operational taxonomic unit (OTU) results. There were no significant differences in alpha diversity among groups. However, there were fewer total species in the TW group than in the NW and OB groups (Figure 2). PCoA and Beta diversity analysis by weighted UniFrac distance analysis revealed significant differences in the composition and abundance of intestinal microorganisms among the three groups (Figures 3, 4).

**Figure 5:**
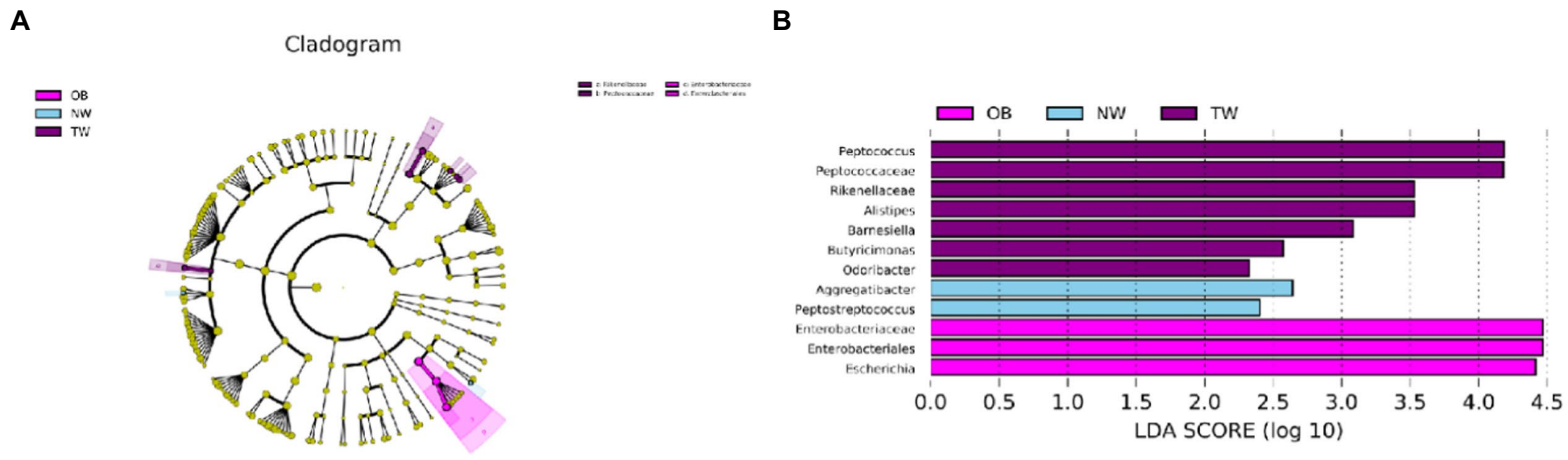
Taxonomic differences in the microbial 16S rRNA gene by a LEfSe analysis. **(A)** Differences in gut bacterial taxa, from the phylum level to the species level, are represented by a cladogram (red indicates taxa enriched in the non-overweight group and green indicates taxa enriched in the overweight group). **(B)** Histogram of taxa (using LDA > 2.0) in each group.

The compositions of intestinal microorganisms in the three groups were analyzed by LEfSE. *Enterobacteriaceae* and *Enterobacteriaies* were significantly enriched in the OB group, *Peptococcus* and *Butyricimonas* were significantly enriched in in the NW group, and *Aggregatibacter* and *PeptoStreptococcus* were significantly enriched in the TW group (Figure 5).

**Figure 6:**
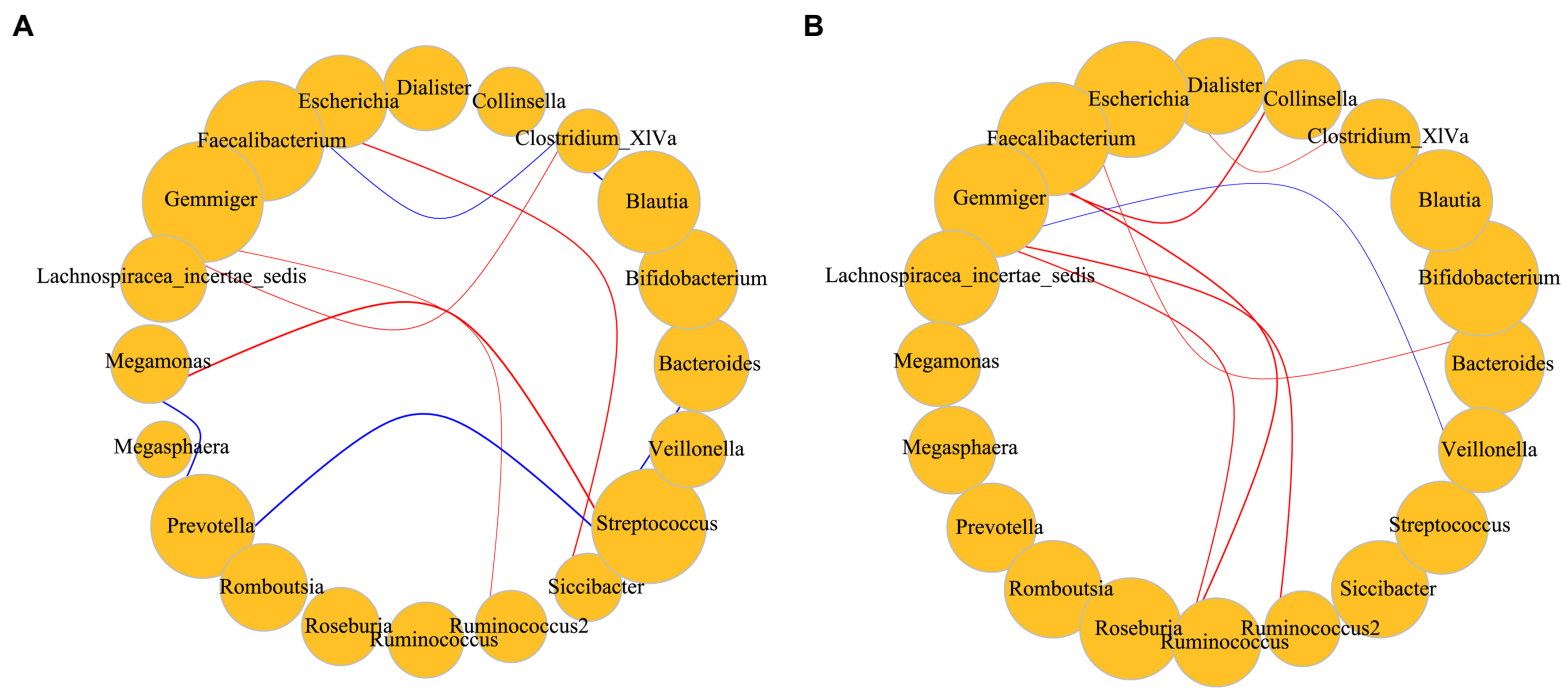
**(A)** Correlations among dominant bacteria in subjects with BMI < 28; **(B)** Correlations among dominant bacteria in subjects with BMI > 28. Red represents a positive correlation; blue represents a negative correlation. Size represents the relative abundance of the genus, including only dominant bacteria. The *p* value for significant correlations are less than 0.05 and the absolute value of the correlation distance calculated by the Spearman method is greater than 0.45. Correlation of the gut microbiota was analyzed using the Hmisc package and igraph package in R v4.1.3.

The population was divided into two groups using BMI = 28 as a threshold. Only the dominant bacteria with high correlations were screened and compared among groups. Predominant bacteria in obese individuals were frequently positively correlated; in contrast, there were more significant negative correlations among taxon abundances in individuals with BMI < 28 (Figure 6).

### 3.4. LPS determination

**Figure 7:**
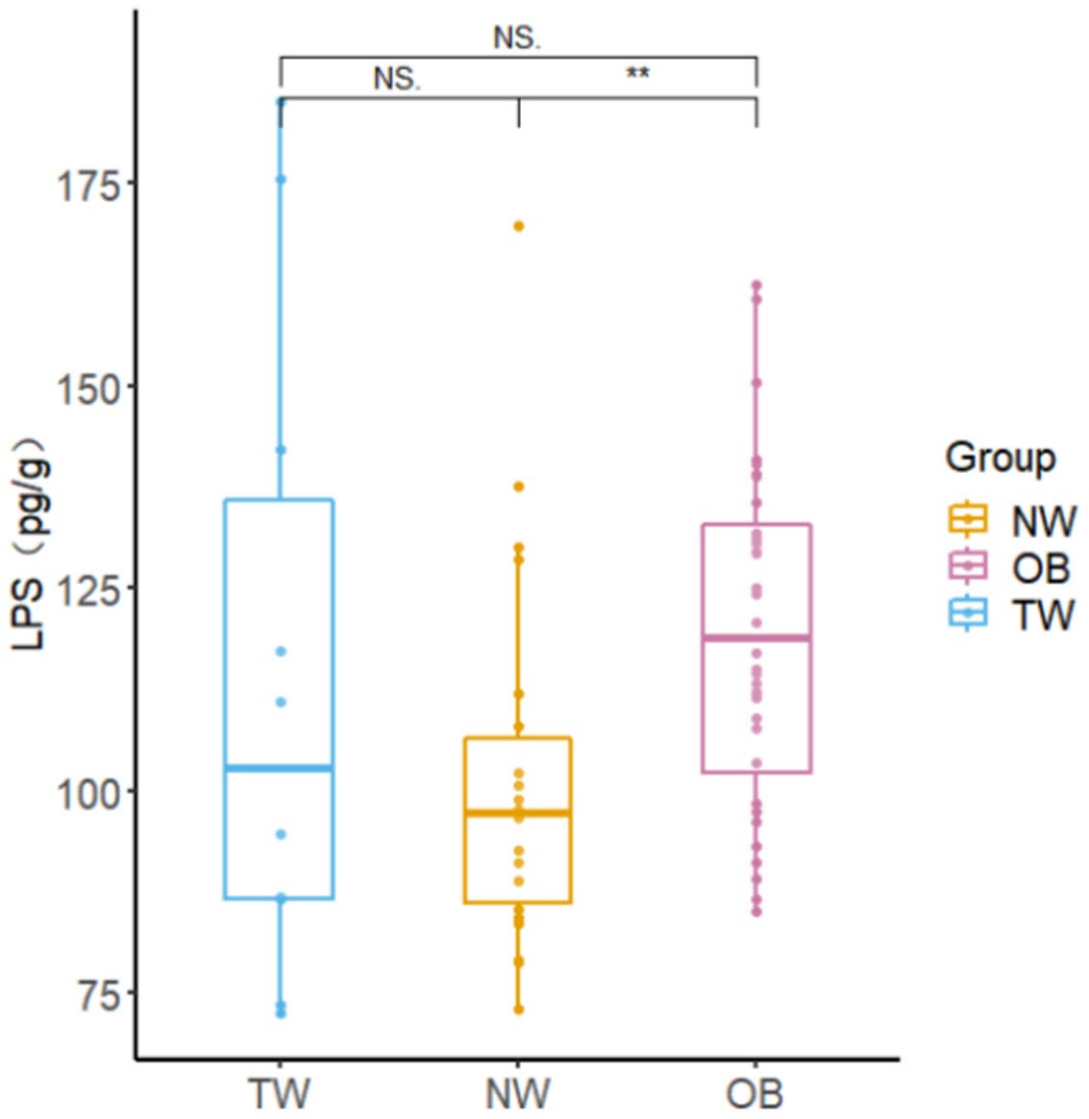
Fecal LPS contents in different groups. Box-and-whisker plots represent the median, interquartile range, and minimum and maximum LPS content within each group. Asterisks above the boxes indicate significant differences (*p* < 0.05). *t*-test was used to test for significant differences in statistics. ^NS^*p* > 0.05, ^*^p ≤ 0.05, and ^**^p ≤ 0.01.

The intestinal LPS content in young college students was 20–180 pg./g, which was higher than the intestinal LPS content in the NW group. There was a significant difference (*p* = 0.004 < 0.05) in LPS contents between the NW group and OB group (Figure 7).

### 3.5. Differential analysis of SCFA contents in different populations

**Figure 8:**
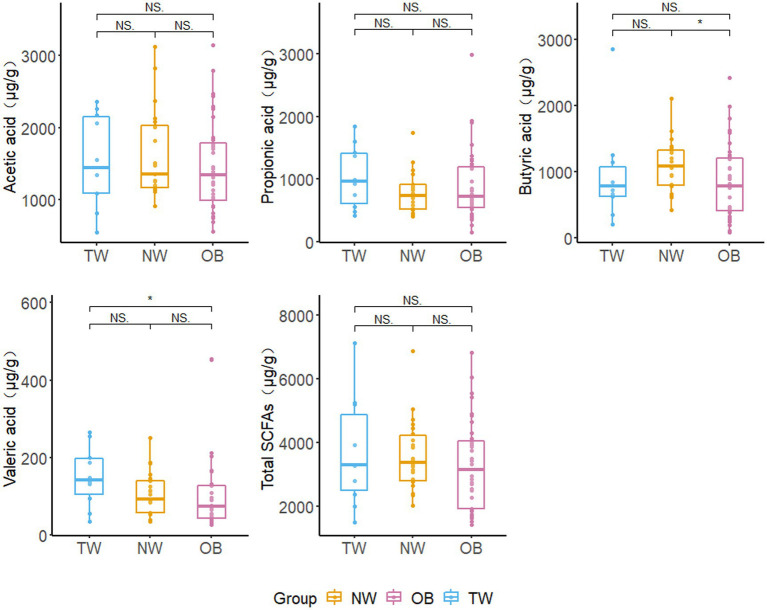
Fecal SCFA contents in different groups. Box and whisker plots represent the median, interquartile range, and minimum and maximum values for the SCFA content in each group. Asterisks above the boxes indicate significant differences (*p* < 0.05). *t*-test was used to test for significant differences in statistics. ^NS^*p* > 0.05, ^*^p ≤ 0.05, and ^**^p ≤ 0.01.

The low isobutyric acid and isovaleric acid contents in the samples did not reach the quantitative limit of detection. Accordingly, these were excluded from subsequent analyses. There were no significant differences in the total SCFA content among the three groups with different obesity statuses; however, the average total SCFA content in the NW group was higher than that in the OB group. The butyric acid content in NW group was significantly higher (*p* = 0.028 < 0.05) than that in the OB group. However, there was no significant difference in the butyric acid content between the TW group and OB group. The valeric acid content was highest in the TW group and was significantly higher (*p* = 0.04 < 0.05) in the TW group than in the OB group (Figure 8).

### 3.6. Correlations among SCFA, obesity indicators, and gut microbes

**Figure 9:**
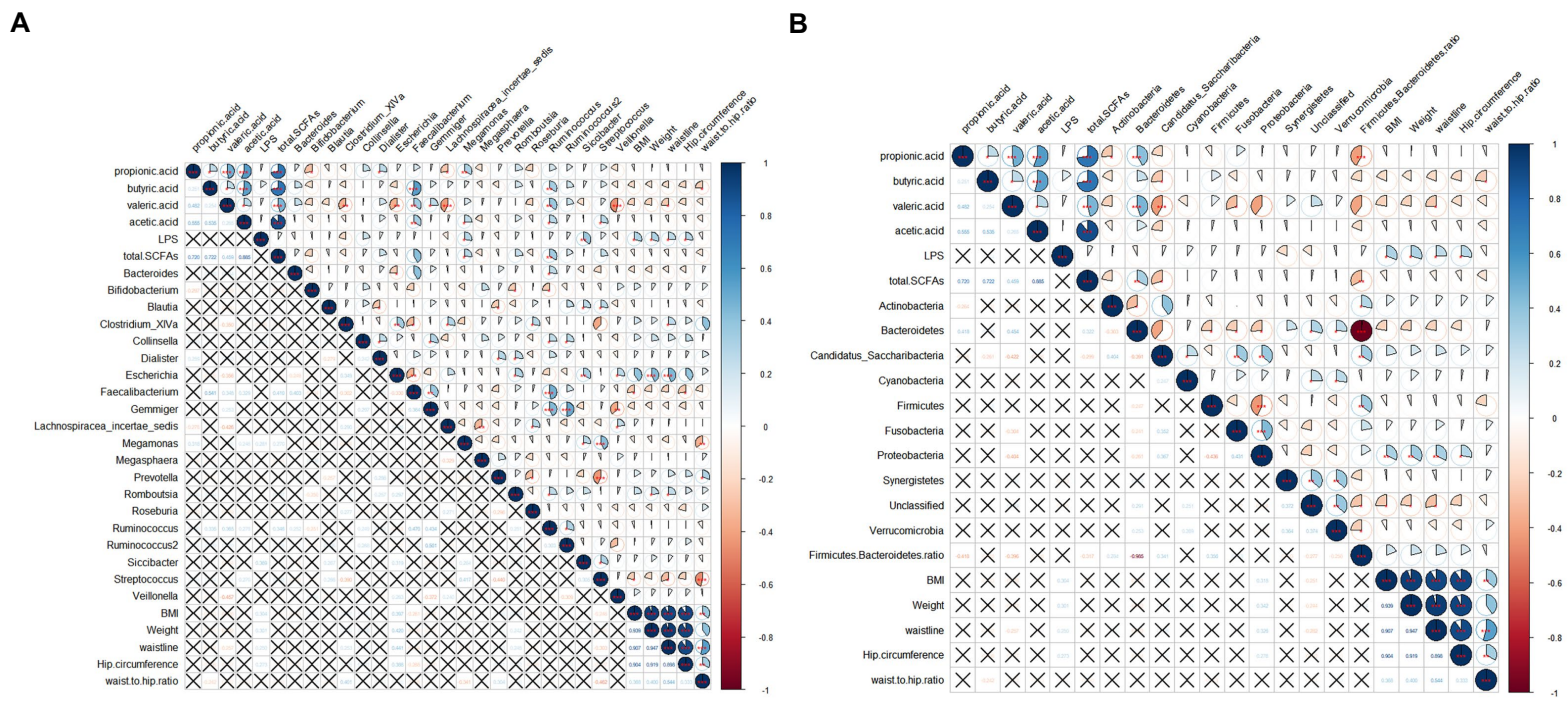
Correlation analysis among obesity indicators, gut microbes, SCFAs, and LPS. **(A)** Correlation between abundances of dominant bacterial genera and other indicators; **(B)** Correlations between abundances of gut microbes at the phylum level and other indicators. The pie chart on the upper right indicates the magnitude of the correlation, with a star indicating significance; the bottom left indicates the correlation coefficient. A cross indicates that the correlation is not significant. Spearman was used to test for correlation.

Correlations among the intestinal flora abundance, metabolic indexes, and physical indexes were evaluated (Figure 9). Both butyrate and propionate contents were negatively correlated with BMI, although the correlations were not significant. There was a significant negative correlation between the butyric acid content and waist-hip ratio. There was a significant negative correlation between the valeric acid content and waist circumference. LPS was positively correlated with BMI, body weight, waist circumference, and hip circumference. BMI showed a significant positive correlation with the abundance of *Escherichia* and a significant negative correlation with the abundances of *Faecalibacterium* and *Streptococcus*. It is worth noting that the abundance of *Bifidobacterium* was positively correlated with physical indicators, such as BMI, although the correlations were not significant.

## 4. Discussion

Obesity had become a major public health problem in China, where overweight and obesity have increased rapidly (30). The obesity index during the youth period greatly affects the obesity status and risk of chronic diseases during adulthood (31). College students mostly live on campuses, with meals and accommodation provided by the college, and thus have similar daily diet and routine. Compliance is generally high among college students, and the requirements set by experimenters are supported and met. Therefore, the target population was well-suited for our study. Gut microbes contribute to the development of obesity; however, the mechanism linking gut microbes to obesity is still unclear (16). Various factors can lead to changes in the gut microbiota, in turn affecting fat distribution (32). In this study, the gut microbial diversity and contents of SCFAs and LPS in fecal samples from 68 college students recruited from Shijiazhuang City, Hebei Province, China were analyzed to explore the associations among gut microbes, metabolites, and obesity in young adults. We found differences in gut microbes and metabolites among people with different BMIs. BMI was significantly correlated with LPS content, while SCFA content was not significantly correlated with BMI and LPS content.

The abundance of Faecalibacterium was negatively correlated with various physical indicators related to obesity. Faecalibacterium is one of the most important bacterial taxa in the human gut flora, accounting for 5–15% of the total number of bacteria detected in fecal samples from healthy individuals (33). However, conclusive studies indicating that *Faecalibacterium* is a marker of obesity or is causally related to obesity are lacking. This result was consistent with those of a study in Iran showing a negative correlation between *Faecalibacterium* abundance and body metrics (34). However, conflicting results have been reported. For example, a study has shown that the *Faecalibacterium* abundance in obese children in southern India was significantly higher than that in non-obese children (35). It is possible that a large amount of *Faecalibacterium* in the gut will increase energy recovery from unabsorbed carbohydrates, which would not contribute to dietary energy intake. *Faecalibacterium* is the main butyrate-producing bacterium (36). The abundance of *Faecalibacterium* had a significant positive correlation with the butyric acid content in feces. *Faecalibacterium* inhibits IL-8 production by producing butyrate to block NF-κB activation, a key anti-inflammatory pathway (37). In fact, an inverse correlation between *Faecalibacterium* abundance and pathological responses in inflammatory disease has been reported (38). Although the exact mechanism of action of *Faecalibacterium* in the gut has not been determined, research generally supports the correlation between the abundance of the genus and obesity indicators (39).

The abundance of *Bifidobacterium* was positively correlated with obesity indicators, inconsistent with the results of previous studies. *Bifidobacterium* is one of the most abundant bacteria in the gut of healthy individuals in early life and plays an important role in gut homeostasis and immune system development (40). The *Bifidobacterium* abundance and species diversity also decrease in late adulthood and in some diseases (41). In general, a higher proportion of *Bifidobacterium* in the gut is considered beneficial for health. Some previous studies have shown that *Bifidobacterium* can promote weight loss. A lower body weight is related to a higher abundance of *Bifidobacterium*, and *Bifidobacterium* could be used as a probiotic to promote weight loss (42, 43). In intervention studies, *Bifidobacterium* significantly reduced the body weight, BMI, waist circumference, and fat content of adults (44). However, there is also evidence that the proportion of *Bifidobacterium* in the intestine of individuals with obesity is increased (45). This can be explained by variation among populations or small sample sizes. There are many explanations for the inconsistent results among gut microbiota studies, including differences in living environment, diet, and lifestyle. Therefore, it is necessary to identify commonalities and differences in the gut microbiome in different populations over a small range while controlling for these factors.

Both butyric acid and propionic acid contents were negatively but not significantly correlated with BMI. SCFAs have emerged as key determinants of host health and disease, with recent evidence that they can modify the epigenome (46). This result is contrary to those of a study in India, in which the fecal butyrate concentration was elevated in individuals with obesity, even in comparisons of groups with similar diets (47). However, cross-sectional data have shown that there is an inverse relationship between fasting plasma butyrate and BMI, suggesting that circulating butyrate levels are reduced in obesity (48). Therefore, higher fecal butyrate levels in the obese group may simply reflect differences in absorption or microbial utilization, rather than increased production.

The metabolism of SCFAs by the host can increase energy obtained from the diet, which may contribute to obesity, depending on the total caloric intake. However, several studies of rodents have suggested that SCFAs prevent obesity by increasing energy expenditure and appetite control (49). Propionate could affect weight control *via* sympathetic nervous system activity. The effect of butyric acid on obesity needs to be further confirmed (22). Butyrate may reduce obesity symptoms by improving insulin resistance and regulating the production and metabolism of gastrointestinal hormones and growth hormones (50). However, *in vitro* and *in vivo* studies have shown that butyrate can trigger adipocyte proliferation by stimulating adipogenesis (51). Therefore, additional research is needed to understand the apparently contradictory effects of butyrate on obesity.

We detected significant differences in the gut-derived LPS content in fecal samples of college students with different degrees of BMI. LPS had a significant positive correlation with BMI. Under standardized conditions, there are differences in the ability of LPS of different gram-negative bacteria to produce inflammatory responses in cultured cells (52). LPS plays a central role in the intestinal innate immune response and can be absorbed through intercellular junctions, a process that can be regulated by factors that control intestinal permeability. The number and composition of gram-negative bacteria in the intestinal tract are important determinants of the systemic availability of LPS (28). Evidence suggests that a systemic low level of gut-derived LPS might play an important role in obesity-related mechanisms (53). Obesity is accompanied by a chronic low-grade inflammatory state characterized by systemic and local elevations of proinflammatory cytokines and acute phase proteins (54). Various studies have reported a significant correlation among elevated LPS, increased inflammatory factors, and fat deposition during obesity (23). It has been reported that there is a significant positive correlation between serum LPS content and BMI (55).

Our research focused on Shijiazhuang university students had several advantages over previous work. The large sample size enabled us to control complex factors, and volunteer recruitment was limited to individuals who do not use drugs, thus limiting the possibility of reverse causality. The limitations of the study include the cross-sectional experimental design, excluding the inference of causality. LPS in feces can only indirectly indicate intestinal permeability and cannot be used to determine serum LPS content. For SCFAs, fecal concentrations may not accurately represent physiological concentrations, as less than 10% of total butyric acid produced is excreted in feces. This suggests that plasma SCFAs may be a better biomarker of the health effects of butyrate metabolism. Because most of the participants did not control their diet before sampling, we could not rule out interference related to diet. Furthermore, since this was an observational study, we could not rule out the contributions of other factors. It is difficult to analyze the causal relationship between the intestinal microbiological composition and physical indicators based on cross-sectional data, and longitudinal studies and studies with larger datasets may further reveal the relationship between intestinal microorganisms and physical indicators of young college students with different degrees of BMI in the future. Additional research is needed to understand the contradictory effects of butyrate on obesity. Further studies should determine the effect of butyric acid on obesity or obesity-related diseases and the effects of obesity on the production and absorption of butyric acid, providing a theoretical basis for clinical trials centered on butyric acid.

We detected significant differences in intestinal microbial diversity among young college students with different body mass indexes and linked these differences with variation in the LPS and SCFA contents in the feces. The LPS content in the feces of obese young college students was significantly higher than that of healthy individuals. The content of butyric acid in obese people was significantly lower than that in healthy people, and the content of valeric acid in obese people was significantly lower than that in the underweight group. These results enrich our understanding of the relationships between intestinal microorganisms, metabolism, and obesity and provide theoretical support for the prevention and treatment of obesity.

## Data availability statement

The original contributions presented in the study are publicly available. This data can be found at: https://www.ncbi.nlm.nih.gov/bioproject/PRJNA884811.

## Author contributions

BS, SZ, and SW conceived and supervised the study. BS, KZ, SZ, YX, XJ, and SW participated in the study design and data collection. BS and SW interpreted the results and provided funding. BS wrote the first draft of the original manuscript. BS, KZ, YX, HL, XJ, and SW provided a critical revision of the manuscript. All authors contributed to the article and approved the submitted version.

## Funding

This study was funded by Innovation Ability Improvement plan project of Hebei (grant number 22567685H) and the Post-subsidy and reward special fund project of Shijiazhuang (grant number 226790217H).

## Conflict of interest

Junlebao Dairy Group Co., Ltd. has hosted and cooperated with us on these projects and provided materials/equipment and funds for the project. SW and YX are employed by Shijiazhuang Junlebao Dairy Co., Ltd., China.

The remaining authors declare that the research was conducted in the absence of any commercial or financial relationships that could be construed as a potential conflict of interest.

## Publisher’s note

All claims expressed in this article are solely those of the authors and do not necessarily represent those of their affiliated organizations, or those of the publisher, the editors and the reviewers. Any product that may be evaluated in this article, or claim that may be made by its manufacturer, is not guaranteed or endorsed by the publisher.

## Footnotes

^1^ http://www.r-project.org

^2^ http://meta.bgi.com/
